# Bioinformatics analysis reveals the potential role of matrix metalloproteinases in immunity and urolithiasis

**DOI:** 10.3389/fimmu.2023.1158379

**Published:** 2023-03-15

**Authors:** Sen-Yuan Hong, Hong-Cheng Jiang, Wen-Chao Xu, He-Song Zeng, Shao-Gang Wang, Bao-Long Qin

**Affiliations:** ^1^ Department of Urology, Tongji Hospital, Tongji Medical College, Huazhong University of Science and Technology, Wuhan, China; ^2^ Division of Cardiology, Department of Internal Medicine, Tongji Hospital, Tongji Medical College, Huazhong University of Science and Technology, Wuhan, China

**Keywords:** urolithiasis, bioinformatics, MMPs, immune infiltration, macrophages

## Abstract

**Background:**

The pathogenesis of urolithiasis remains unclear, making the development of medications for treatment and prevention stagnant. Randall’s plaques (RPs) begin as interstitial calcium phosphate crystal deposits, grow outward and breach the renal papillary surface, acting as attachment for CaOx stones. Since matrix metalloproteinases (MMPs) can degrade all components of extracellular matrix (ECM), they might participate in the breach of RPs. Besides, MMPs can modulate the immune response and inflammation, which were confirmed to be involved in urolithiasis. We aimed to investigate the role of MMPs in the development of RPs and stone formation.

**Methods:**

The public dataset GSE73680 was mined to identify differentially expressed MMPs (DEMMPs) between normal tissues and RPs. WGCNA and three machine learning algorithms were performed to screen the hub DEMMPs. *In vitro* experiments were conducted for validation. Afterwards, RPs samples were classified into clusters based on the hub DEMMPs expression. Differentially expressed genes (DEGs) between clusters were identified and functional enrichment analysis and GSEA were applied to explore the biological role of DEGs. Moreover, the immune infiltration levels between clusters were evaluated by CIBERSORT and ssGSEA.

**Results:**

Five DEMMPs, including MMP1, MMP3, MMP9, MMP10, and MMP12, were identified between normal tissues and RPs, and all of them were elevated in RPs. Based on WGCNA and three machine learning algorithms, all of five DEMMPs were regarded as hub DEMMPs. *In vitro* validation found the expression of hub DEMMPs also increased in renal tubular epithelial cells under lithogenic environment. RPs samples were divided into two clusters and cluster A exhibited higher expression of hub DEMMPs compared to cluster B. Functional enrichment analysis and GSEA found DEGs were enriched in immune-related functions and pathways. Moreover, increased infiltration of M1 macrophages and enhanced levels of inflammation were observed in cluster A by immune infiltration analysis.

**Conclusion:**

We assumed that MMPs might participate in RPs and stone formation through ECM degradation and macrophages-mediated immune response and inflammation. Our findings offer a novel perspective on the role of MMPs in immunity and urolithiasis for the first time, and provide potential biomarkers to develop targets for treatment and prevention.

## Introduction

Urolithiasis is a common urological disease with an increasing incidence rate and a high recurrence rate (1). Recurrent episodes of stones lead to renal injury and chronic kidney disease, posing a huge economic burden on individuals and society (2). Although rapid advances of surgical techniques have greatly improved the stone removal efficiency, the development of medical drugs to treat and prevent urolithiasis has stalled, as its pathogenesis is not fully understood. Calcium oxalate (CaOx) stones are the most common stone type (3), and hypercalciuria and hyperoxaluria are major risk factors for stone formation (4). Randall’s plaques (RPs) theory is the most widely accepted theory about CaOx stone formation. RPs begin as calcium phosphate (CaP) crystal deposits in the renal interstitium, grow and extend outwards reaching the renal papillary surface, and become exposed to the pelvis urine. CaOx crystals could attach to the exposed sites and grow into CaOx stones (5, 6). Apparently, RPs are subepithelial deposits that must have their surfaces breached. Some researchers believe that papillary surface epithelium can be breached through the involvement of matrix metalloproteinases (MMPs) and/or the sheer force of the growing plaque (7). Still, how such a breach happens remain obscure. MMPs are a family of zinc and calcium dependent endopeptidases which are able to degrade all components of the extracellular matrix (ECM) and process various bioactive substances (8). Currently, the MMP family consists of 23 members in human, which can be divided into 6 major types, including collagenases, gelatinases, stromelysins, matrilysins, membrane-type MMPs, and other MMPs (8). Under physiological conditions, MMPs participate in tissue repair and remodelling, morphogenesis, wound healing, cell proliferation and migration (9, 10). Dysregulation of MMPs might result in tissue destruction and homeostasis disorders and contribute to various diseases, such as cancer, atherosclerosis, aortic aneurysms, arthritis, tissue ulcers and fibrosis (9, 10). In addition, MMPs have been implicated in a wide range of kidney diseases, such as kidney fibrosis, glomerular disease, and diabetic kidney disease (11). So far, few studies have been reported the relationship between MMPs and urolithiasis.

Besides, immunity and inflammation were tightly associated with urolithiasis (6). Immune cells and pro-inflammatory molecules were shown to be increased in RP tissues and macrophages seem to participate critically in stone formation (12). M1 macrophages can induce inflammatory response to accelerate renal injury and CaOx crystals depositions, while M2 macrophages can phagocytose and degrade crystals (13). MMPs were also found to be involved in immunity and inflammation, serving as modulators of immune response and inflammatory processes, which can influence and shaped the phenotype of immune cells (14). Therefore, we assumed that MMPs might play a pivotal role in the development of RPs. However, the precise mechanism is still a mystery.

In this study, we first used the dataset GSE73680 to identify differentially expressed MMPs (DEMMPs) between normal renal papillary tissues and RPs. Then, we performed weighted gene co-expression network analysis (WGCNA) and three machine learning algorithms to screen the hub DEMMPs. Afterwards, we divided RPs samples into two clusters based on the hub DEMMPs expression pattern, identified differentially expressed genes (DEGs) between them and conducted further functional enrichment analysis and gene set enrichment analysis (GSEA). Moreover, we evaluate the immune infiltration levels between two clusters. We believe our findings will offer a novel insight into the role of MMPs in urolithiasis and provide potential biomarkers to develop therapeutic targets for the disease.

## Materials and methods

### Acquisition and processing of data

**Figure 1:**
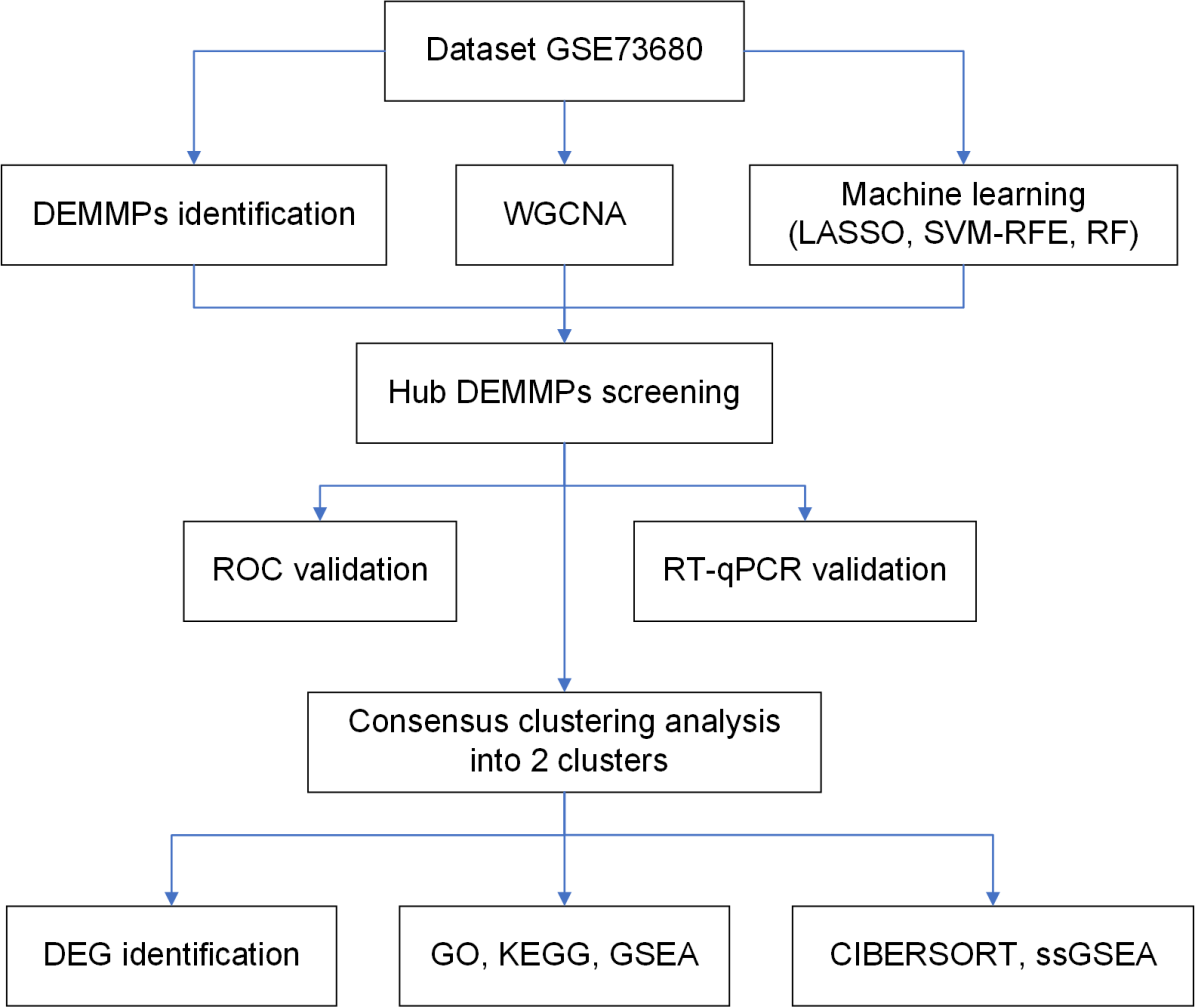
The flowchart of the present study.

The flowchart of the study is shown in **Figure 1**. The dataset GSE73680 was obtained from gene expression omnibus database (GEO, http://www.ncbi.nlm.nih.gov/geo/), which contains 6 normal renal papillary tissues from non-stone formers and 29 RPs from stone formers (12). The annotation information provided in the platform GPL17077 were used to transform the probe IDs into gene symbols.

### Identification of DEMMPs

The MMP genes were obtained through HUGO Gene Nomenclature Committee (https://www.genenames.org/). The Wilcox test was applied to identify DEMMPs between normal tissues and RPs, and the results were shown as a boxplot using the “ggpubr” package. Besides, the correlations between MMPs were calculated by Spearman correlation analysis and the correlation matrices were visualized using the “corrplot” package. The protein-protein interaction (PPI) network of MMPs was established by the STRING database (https://string-db.org/) with minimum interaction score ≥ 0.4. We then calculate the sides of each node and sort genes based on the rank of the connection number of each gene.

### Weighted gene co-expression network analysis

The “WGCNA” package was utilized to construct the co-expression networks for identification of hub module related with urolithiasis (15). Initially, the Pearson’s correlation matrices were performed for all pair-wise genes and a weighted adjacency matrix was constructed with the formula a_mn_ = |c_mn_|^β^ (c_mn_ = Pearson’s correlation between gene m and gene n; a_mn_ = adjacency between gene m and gene n). Then, the soft threshold power value, parameter β was calculated, which emphasized strong correlations between genes and penalize weak correlations. A suitable parameter β was applied to construct a scale-free network. Next, the weighted adjacency matrix was transformed into a topological overlap measure (TOM) matrix, which could measure the network connectivity of a gene defined as the sum of its adjacency with all other genes for the network generation (16). Afterwards, average linkage hierarchical clustering was performed to classify genes with similar expression profiles into the same gene modules based on the TOM‐based dissimilarity measure with a minimum gene group size of 50 for the genes dendrogram (17). Finally, we calculated the gene significance (GS), module membership (MM), and associated modules with clinical traits to identify the urolithiasis-related modules with the highest coefficient square (R2) and the P-value < 0.05. We took the intersection between DEMMPs and genes in urolithiasis-related modules to obtain the hub DEMMPs

### Machine learning

Three machine learning algorithms were used to screen the hub DEMMPs. The least absolute shrinkage and selection operator (LASSO) is a regression approach used for dimensionality reduction and feature selection to increase predicted accuracy and model comprehensibility (18), which was performed by the “glmnet” package (19). Support vector machine recursive feature elimination (SVM-RFE) is a powerful method to rank features and to select the significant ones for classification (20), which was performed by the “kernlab” and “e1071” packages (21). Random forest (RF) is an ensemble learning method for classification by constructing a multitude of decision trees at training time (22), which was performed by the “randomForest” package (23). DEMMPs identified more than three times in the above four analysis (WGCNA, LASSO, SVM-RFE, RF) were considered as hub DEMMPs. The receiver operating characteristic (ROC) was constructed and the area under the ROC curve (AUC) was calculated to evaluate the capacity of hub DEMMPs to discriminate RPs from normal tissues using the “pROC” package (24).

### Cell experiments and validation of hub DEMMPs

The normal rat kidney proximal tubular epithelial cell line (NRK-52E) was obtained from the Type Culture Collection of the Chinese Academy of Sciences (Shanghai, China). Cells were cultured in Dulbecco’s modified eagle medium (DMEM; Hyclone, USA) with 10% fetal bovine serum (FBS; Gibco, USA) at 37°C in a humidified atmosphere with 5% CO_2_. The normal human kidney proximal tubular epithelial cell line (HK-2) was purchased from Procell (Wuhan, China) and cultured in DMEM-F12 medium (Hyclone, USA) with 10% FBS at 37°C in a humidified atmosphere with 5% CO2. NRK-52E and HK-2 cells were treated with oxalate (1 mmol/L) for 24 h, and cells without treatments were used as a control group.

Total RNA was extracted using a TRIzol reagent (Vazyme, Nanjing, China). Then, the purified RNA was reverse-transcribed into cDNA using a cDNA synthesis kit (Yeasen, Shanghai, China). Next, real-time quantitative polymerase chain reaction (RT-qPCR) was performed using SYBR Green Master Mix reagent (Yeasen, Shanghai, China) on the PCR System. The mRNA expression levels of genes were calculated using the 2^-ΔΔCt^ method. The sequences of primers used in this study were presented in **Table 1**.

**Table 1:** Sequences of primers used RT-qPCR.

Gene	Primer	Sequence (5'-3')	Product length
GAPDH (Rat)	Forward	ACAGCAACAGGGTGGTGGAC	250
	Reverse	TTTGAGGGTGCAGCGAACTT	
MMP1 (Rat)	Forward	GCTTAGCCTTCCTTTGCTGTTGC	136
	Reverse	GACGTCTTCACCCAAGTTGTAGTAG	
MMP3 (Rat)	Forward	CCACAGAATCCCCTGATGTC	103
	Reverse	CTGACTGCATCGAAGGACAA	
MMP9 (Rat)	Forward	GCTGGGCTTAGATCATTCTTCAGTG	109
	Reverse	CAGATGCTGGATGCCTTTTATGTCG	
MMP10 (Rat)	Forward	TGGTTCCTGTGCCCTCTGTCTC	148
	Reverse	TCGGGATTCCACTGGGTTCTACG	
MMP12 (Rat)	Forward	GGCAACTGGACACCTCAACTCTG	107
	Reverse	CCGCTTCATCCATCTTGACCTCTG	
β-actin (human)	Forward	GTCATTCCAAATATGAGATGCGT	105
	Reverse	TGTGGACTTGGGAGAGGACT	
MMP1 (human)	Forward	CTGGGAGCAAACACATCTGACCTAC	93
	Reverse	TGGAAGGCTTTCTCAATGGCATGG	
MMP3 (human)	Forward	GACAAAGGATACAACAGGGAC	81
	Reverse	GCTGAGTGAAAGAGACCCA	
MMP9 (human)	Forward	AGACCTGGGCAGATTCCAAAC	94
	Reverse	CGGCAAGTCTTCCGAGTAGT	
MMP10 (human)	Forward	GAGATGCCAGCCAAGTGTGATCC	126
	Reverse	AAATTCAGGTTCAGGGTTCCAGTGG	
MMP12 (human)	Forward	GATCCAAAGGCCGTAATGTTCC	86
	Reverse	TGAATGCCACGTATGTCATCAG	

### Consensus clustering analysis

We used the “ConsensusClusterPlus” R package (25) to conduct an unsupervised hierarchical clustering analysis on 29 RPs samples based on the mRNA expression of hub DEMMPs. We used consensus matrix plots, consensus cumulative distribution function (CDF) plots, and the relative change in area under the CDF curve to determine the optimal number of clusters. After that, principal component analysis (PCA) between clusters was performed to observe the differences between clusters. The “limma” package was then used to identify DEGs between clusters with the threshold of |log2(fold change)|>2 and the P-value < 0.05. DEGs were visualized as a volcano plot and a heatmap.

### Functional enrichment analysis and GSEA

To further predict the biological functions of DEGs, Gene Ontology (GO) enrichment analysis and Kyoto Encyclopedia of Genes and Genomes (KEGG) pathway analysis were performed using the “clusterProfiler” package (26). The adjusted P-value < 0.05 was the threshold. We also applied GSEA to explore the significant functional differences between clusters using the “clusterProfiler” package. The gene sets “c5.go.v2022.1.Hs.symbols.gmt” and “c2.cp.kegg.v2022.1.Hs.symbols.gmt” were obtained from the GSEA website MsigDB database (http://www.gsea-msigdb.org/gsea/msigdb/). Gene sets were considered as significantly enriched by False discovery rate (FDR) < 0.25, the adjusted P-value < 0.05 and |normalized enrichment score (NES)| > 1.

### Immune infiltration analysis

We used two methods to investigate the immune infiltration levels between two clusters. CIBERSORT (https://cibersortx.stanford.edu/) was applied to evaluate infiltration status of 22 distinct immune cells in two clusters, and the outcomes were visualized as a violin plot using the “vioplot” package. The correlations between the hub DEMMPs and immune cells were evaluated by Spearman correlation analysis and were visualized as lollipop plots. Single sample gene set enrichment analysis (ssGSEA) was conducted to quantify immune infiltration levels by calculating the enrichment scores of 16 immune cells and 13 immune-related functions in two clusters using the “gsva” package (27, 28), and the results were visualized as a boxplot using the “ggpubr” package.

### Statistical analysis

Data analysis and statistical analysis were performed by R software (version 4.1.3) and GraphPad Prism (version 8.0). The statistical difference between two groups were assessed by the Wilcox test or the unpaired t-test. The correlations between the genes were analyzed by a spearman correlation test. The P-value < 0.05 was considered statistically significant.

## Results

### Identification of DEMMPs

**Figure 2:**
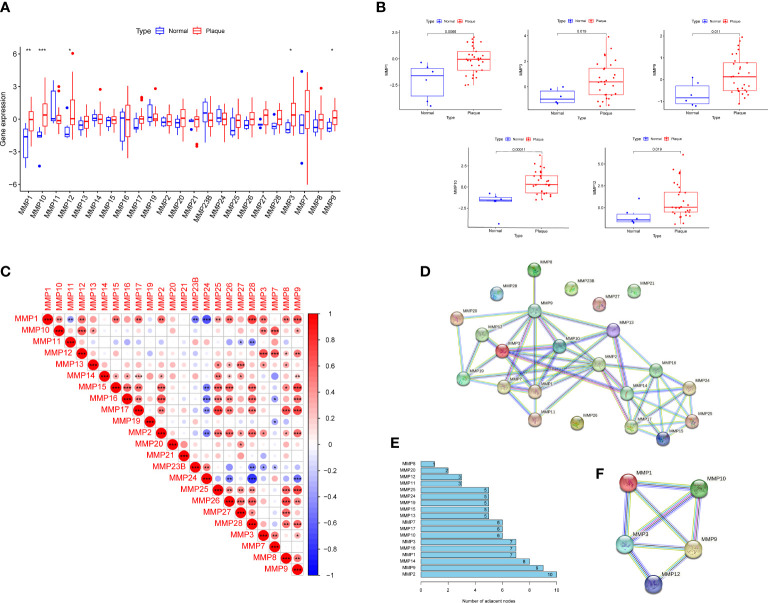
Identification of DEMMPs. **(A)** The boxplot of 26 MMPs expression in normal tissues and RPs. **(B)** The boxplot of five DEMMPs in normal tissues and RPs. **(C)** Correlation analysis between 26 MMPs. **(D)** PPI networks of 26 MMPs. **(E)** The histogram showing the number of adjacent nodes. **(F)** PPI network of five DEMMPs. * represents P < 0.05, ** represents P < 0.01; *** represents P < 0.001.

We first screened DEMMPs between normal tissues and RPs using the Wilcox test. A total of five MMPs, including MMP1, MMP3, MMP9, MMP10, MMP12 were identified as DEMMPs, and all of them were significantly upregulated in RPs (**Figures 2A, B**). Through the correlation analysis, we found all DEMMPs were positively correlated with each other (**Figure 2C**). The PPI network was conducted to investigate the interactions between MMPs at the protein level (**Figures 2D, E**). For DEMMPs, MMP9 have the highest numbers of interacted proteins, followed by MMP1, MMP3, MMP10, and MMP12. Besides, the interactions between DEMMPs were shown in **Figure 2F**.

### Screening of hub DEMMPs *via* WGCNA

**Figure 3:**
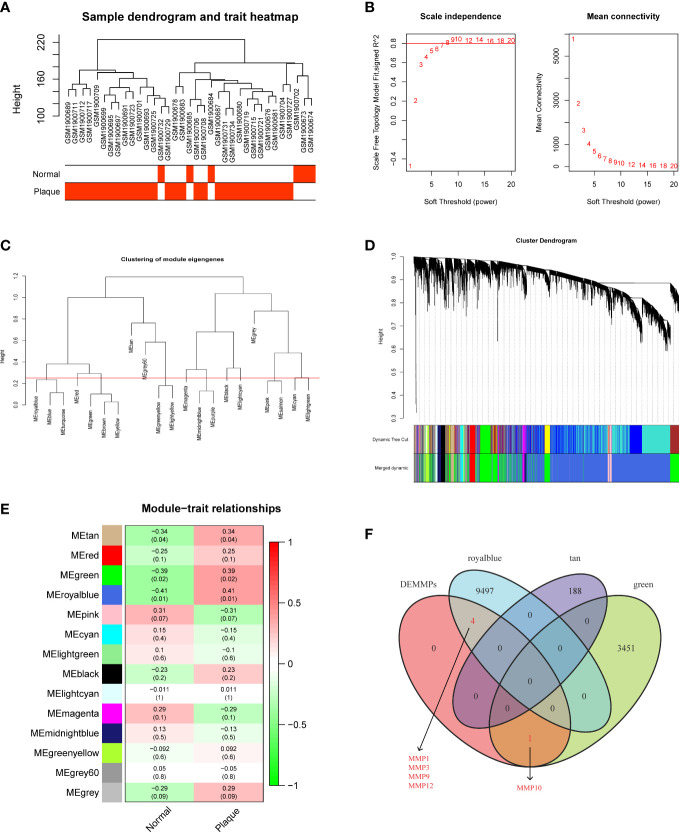
Screening of hub DEMMPs *via* WGCNA. **(A)** Hierarchical clustering tree of 6 normal tissues and 29 RPs gene expression patterns. **(B)** Identification of power value. The red line represents R2 > 0.8 when the power value β is 8. **(C)** Module eigengene dendrogram presented the relationship of the modules generated by the clustering analysis. **(D)** Clustering dendrogram and merging of the gene co-expression modules. Each color represents one module. **(E)** The heatmap of the correlation between modules and clinical traits. The correlation coefficient and P value between the module and clinical traits are shown at the row-column intersection. **(F)** The Venn plot showing the intersection between DEMMPs and the genes in urolithiasis-related modules.

We performed WGCNA to explore modules associated with urolithiasis. The samples were clustered hierarchically to remove outliers, and no outliers were detected and removed in this study (**Figure 3A**). Next, we chose β = 8 as the soft threshold power value to make the scale-free R2 reach 0.8 and construct a scale-free network (**Figure 3B**). A hierarchical clustering tree was constructed and modules were detected by dynamic tree cutting, and modules with highly correlated eigengenes were merged (minimal module size = 50, merge height = 0.25) (**Figure 3C**). A total of 14 color modules were determined (**Figure 3D**). Among them, the royalblue module (r = 0.41, P = 0.01), the green module (r = 0.39, P = 0.02) and the tan module (r = 0.34, P = 0.04) showed significantly positive correlations with urolithiasis (**Figure 3E**). Subsequently, we extracted DMMMPs and genes in urolithiasis-related modules for intersection and found MMP1, MMP3, MMP9, MMP12 were in the royalblue module and MMP10 were in the green module, suggesting all of DEMMPs might be implicated in RPs and urolithiasis (**Figure 3F**).

### Screening of hub DEMMPs *via* machine learning

**Figure 4:**
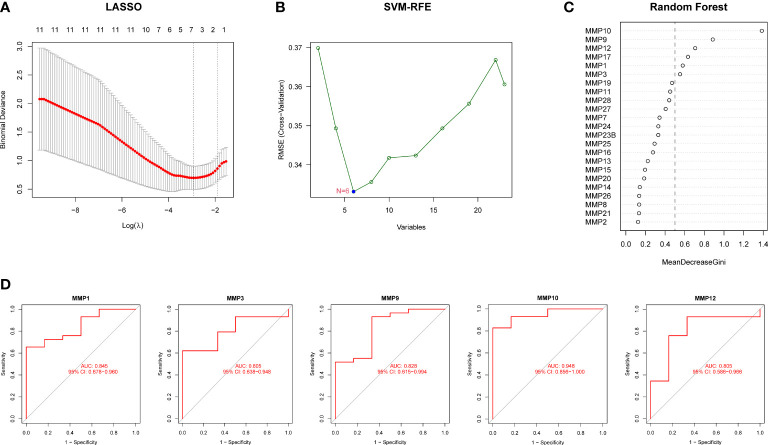
Screening of hub DEMMPs *via* machine learning. **(A)** LASSO analysis. Vertical dashed lines are plotted at the best lambda. **(B)** SVM-RFE algorithm for feature gene selection. **(C)** Ranking of the relative importance of genes in the RF classifier. **(D)** ROC curve of hub DEMMPs in urolithiasis diagnosis.

We further performed three machine learning algorithms using the expression profiles 26 MMPs to identify potential MMPs associated with RPs and urolithiasis. For LASSO regression analysis, six variables, MMP1, MMP3, MMP9, MMP10, MMP27 and, MMP28, were screened as diagnostic markers for urolithiasis (**Figure 4A**). For the SVM-RFE algorithm, the error was minimized when the number of variables was six, including MMP10, MMP17, MMP9, MMP12, MMP1, and MMP3 (**Figure 4B**). For RF algorithm, six candidate genes with relative importance scores greater than 0.5 were identified, including MMP10, MMP9, MMP12, MMP17, MMP1, and MMP3 (**Figure 4C**). Combined with the outcomes of WGCNA, we found all of DEMMPs were identified to be associated with urolithiasis by at least three out of four approaches used. Therefore, we considered all of DEMMPs as hub DEMMPs for further analysis.

Next, we evaluated the diagnostic performance of hub DEMMPs by constructing ROC curves. Notably, MMP10 had the highest AUC among the seven hub genes, with a value of 0.948. The AUC values for other genes were 0.845 for MMP1, 0.828 for MMP9, 0.805 for MMP3, and 0.805 for MMP12, respectively (**Figure 4D**). These results indicated that all five gene signatures had powerful diagnostic values.

### Validation of hub DEMMPs

**Figure 5:**
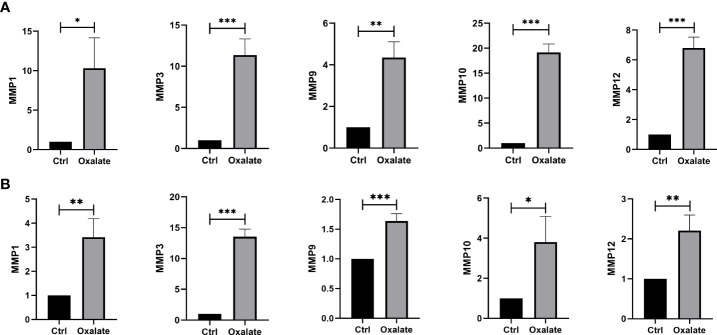
Validation of hub DEMMPs. **(A)** The expression of hub DEMMPs in NRK-52E cells after exposure to oxalate for 24h. **(B)** The expression of hub DEMMPs in HK-2 cells after exposure to oxalate for 24h. * represents P < 0.05, ** represents P < 0.01; *** represents P < 0.001.

We built a cell model of urolithiasis to validate hub genes. NRK-52E cells exposed to oxalate showed higher expression of MMP1 (10.30 fold, P = 0.0139), MMP3 (11.36 fold, P = 0.0008), MMP9 (4.35 fold, P = 0.0015), MMP10 (19.15 fold, P < 0.0001), and MMP12 (6.80 fold, P = 0.0002) compare to control (**Figure 5A**). HK-2 cells exposed to oxalate also showed higher expression of MMP1 (3.42 fold, P = 0.0054), MMP3 (13.53 fold, P < 0.0001), MMP9 (1.64 fold, P = 0.0009), MMP10 (3.81 fold, P = 0.0194), and MMP12 (2.21 fold, P = 0.0059) compare to control (**Figure 5B**). The results were consistent with bioinformatics screening.

### Identification of two clusters based on hub DEMMPs

**Figure 6:**
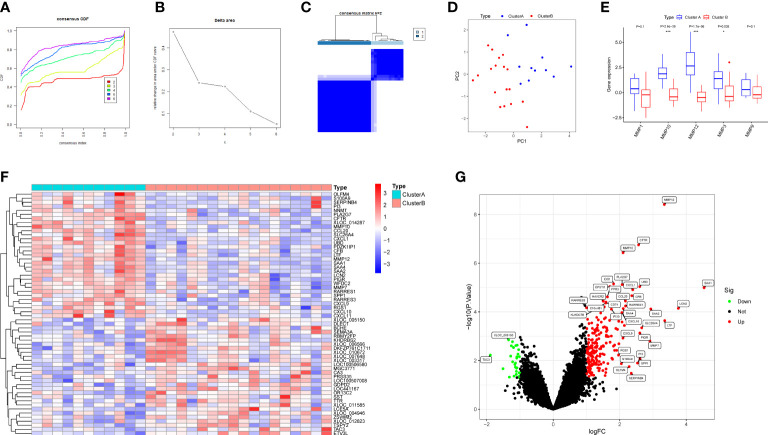
Identification of two clusters based on hub DEMMPs. **(A)** The consensus cumulative distribution function (CDF) plot. **(B)** The relative change in area under the CDF curve. **(C)** The consensus matrix plot when consumption k = 2. **(D)** The PCA plot showing the distribution of two clusters. **(E)** The boxplot of five DEMMPs in cluster A and cluster B **(F)** The heatmap of the top 20 DEGs between two clusters. **(G)** The volcano plot of all DEG between two clusters. * represents P < 0.05, ** represents P < 0.01; *** represents P < 0.001.

RPs samples were clustered by the consensus clustering analysis based on the expression profiles of hub DEMMPs to discover subtypes of urolithiasis. Clustering results are relatively stable when the number of clusters was set to 2 based on the CDF curve and the CDF Delta area curve (**Figures 6A, B**). The consensus matrix plot showed that 29 RPs samples could be divided into two distinct clusters, i.e., cluster A (n = 11) and cluster B (n = 18) (**Figure 6C**). A clear distribution between cluster A and cluster B was presented in the PCA plot (**Figure 6D**). The expression profiles of the hub DEMMPs in the two clusters were visualized as the boxplot (**Figure 6E**). Cluster A showed higher expression of hub DEMMPs compared to Cluster B. Hence, cluster A was considered as high expression group and cluster B was considered as low expression group. A total of 233 DEGs were identified between cluster A and cluster B. Compared to cluster B, 204 DEGs were upregulated and 29 DEGs were downregulated in cluster A (**Figures 6F, G**).

### Functional distinctions between two clusters

**Figure 7:**
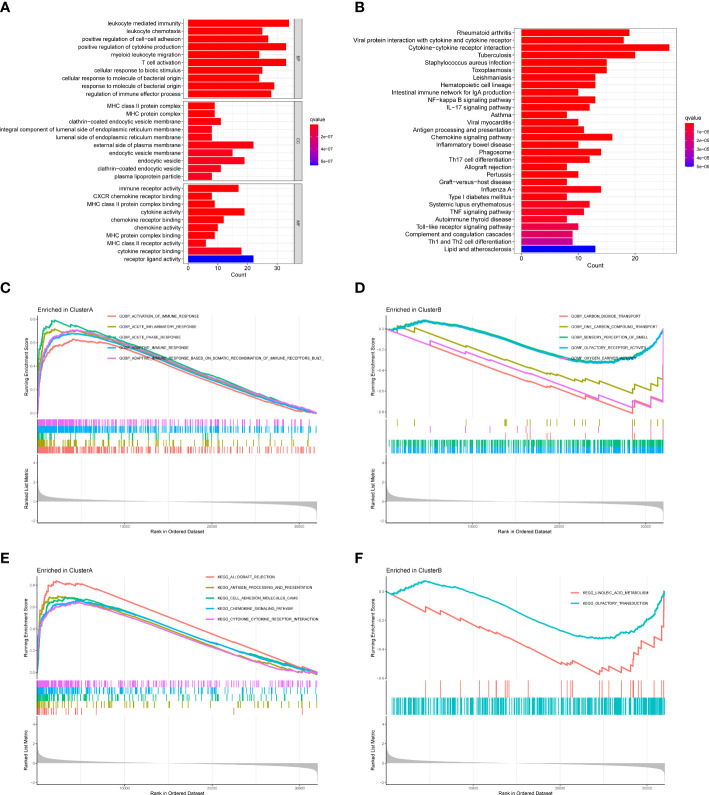
Functional enrichment analysis and GSEA between two clusters. **(A)** GO analysis of DEGs. **(B)** KEGG analysis of DEGs. **(C)** GSEA showing the enriched GO terms in cluster A **(D)** GSEA showing the enriched GO terms in cluster B **(E)** GSEA showing the enriched KEGG pathways in cluster A **(F)** GSEA showing the enriched KEGG pathways in cluster B.

We further applied GO enrichment analysis and KEGG pathway analysis to explore the potential role of DEGs. Among the GO analysis results (**Figure 7A**), leukocyte mediated immunity, leukocyte chemotaxis, positive regulation of cell-cell adhesion, positive regulation of cytokine production, and myeloid leukocyte migration were the five most significant biological processes (BP); MHC class II protein complex, MHC protein complex, and clathrin-coated endocytic vesicle membrane were the three most significant cellular components (CC); immune receptor activity, CXCR chemokine receptor binding, and MHC class II protein complex binding were the three most significant molecular functions (MF). According to the KEGG analysis, DEGs were enriched in rheumatoid arthritis, viral protein interaction with cytokine and cytokine receptor, cytokine-cytokine receptor interaction, and so on (**Figure 7B**).

We also performed GSEA to explore the enriched GO terms and KEGG pathways in two clusters. For GO terms, activation of immune response, acute inflammatory response, acute phase response, and adaptive immune response were enriched in cluster A, while carbon dioxide transport, one carbon compound transport, sensory perception of smell, olfactory receptor activity, and oxygen carrier activity were enriched in cluster B (**Figures 7C, D**). For KEGG pathways, allograft rejection, antigen processing and presentation, cell adhesion molecules cams, chemokine signaling pathway, and cytokine-cytokine receptor interaction were enriched in cluster A, while linoleic acid metabolism and olfactory transduction were enriched in cluster B (**Figures 7E, F**). Since cluster A had higher expressions of hub DEMMPs, our results suggested MMPs might be associated with immune response and inflammation.

### Differences of immune infiltration between two clusters

**Figure 8:**
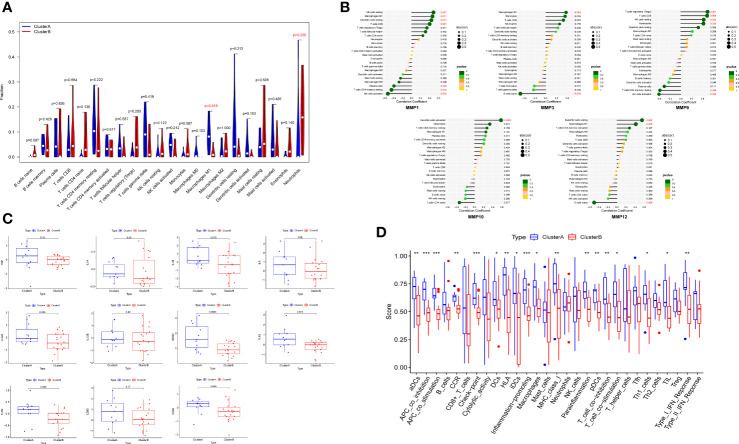
Immune infiltration analysis between two clusters. **(A)** The violin plot showing different proportions of 22 immune cells between two clusters by CIBERSORT. **(B)** The lollipop plots showing the correlations between hub DEMMPs and immune cells. **(C)** The boxplot of 11 M1 macrophages markers in cluster A and cluster B **(D)** The boxplot showing different proportions of 16 immune cells and 13 immune-related functions between two cluster by ssGSEA. * represents P < 0.05, ** represents P < 0.01; *** represents P < 0.001.

Based on differences of functional analysis, we further investigate the relationship between immune infiltration levels between two clusters. The outcomes of CIBERSORT showed that the level of macrophages M1 was relatively high, while the level of neutrophils was relatively low in cluster A compared to cluster B (**Figure 8A**). The correlations between hub DEMMPs and immune cells were shown in **Figure 8B**. Hub DEMMPs exhibited significant positive and negative correlations with different immune cells. MMP1, MMP3, and MMP12 were positively correlated with M1 macrophages (R = 0.46, P = 0.011; R = 0.38, P = 0.044; R = 0.42, P = 0.025). MMP9 and MMP10 showed positive correlation tendency with M1 macrophages, albeit without reaching statistical significance (R = 0.21, P = 0.269; R = 0.25, P = 0.191).

Since cluster A showed increased M1 macrophages levels, we compared markers of M1 macrophages between 2 clusters, including TNF, IL1A, IL1B, IL6, IL12A, IL12B, NOS2, TLR2, TLR4, CD80, and CD86 (**Figure 8C**). The expression of NOS2, TLR2, and TLR4 were higher in cluster A compared to cluster B. All other markers tended to increase in cluster A, although not statistically significant. In addition, we compared ssGSEA scores between normal tissues and RPs and found cluster A generally had higher immune infiltration (**Figure 8D**). For example, for immune cells, macrophages and dendritic cells were more abundant in cluster A; for immune-related functions, inflammation-promoting and type I IFN response were enriched in cluster A.

## Discussion

Development of medications for the treatment and prevention of urolithiasis has been lagging, since its pathogenesis is still unclear. It is well accepted that RPs are the origin of CaOx stones (29). RPs are initially renal interstitial CaP deposits which grow outward and across the renal papillary surface, and become exposed to pelvis urine. CaOx stones will form and develop with the attachment to RPs (5, 6). It is important to break through the renal epithelium during the development of RPs, but the exact mechanism remain an enigma. As MMPs are capable of degrading all kinds of ECM proteins, they might participate in the process of the breach to promote RPs progression. In addition, MMPs are able to modulate the immune response and inflammation, which were confirmed to be involved in urolithiasis (6). Therefore, we aimed to explore the potential role of MMPs in urolithiasis through data mining based on the public gene expression profile of RPs (GSE73680).

We first identified five DEMMPs between normal tissues and RPs, including MMP1, MMP3, MMP9, MMP10, and MMP12, and the expression of all DEMMPs was elevated in RPs. MMP1 belongs to collagenases, MMP3 and MMP10 belong to stromelysins, MMP9 belongs to gelatinases, and MMP12 belongs to other MMPs (8). Correlation analysis showed the expression of all DEMMPs were positively correlated with each other and PPI showed some interactions between them. Studies have demonstrated that MMP1 could cleaves pro-MMP9 into its active form; MMP3 can active pro-MMP1 and pro-MMP9 (10). This might be a good explanation for the results of correlation analysis and PPI.

Four approaches, including WGCNA, LASSO, SVM-RFE, and RF, were performed to screen the hub DEMMPs, and all DEMMPs were identified by at least three out of four approaches used. Therefore, we referred to all DEMMPs as hub DEMMPs. Corresponding AUCs for hub DEMMPs > 0.8 indicated they are powerful biomarkers to distinguish RPs from normal tissues. In addition, we also found that NRK-52E cells upregulated the expression of hub DEMMPs after exposure to oxalate. We assumed that MMPs expression might increase in renal tubular epithelial cells under lithogenic environment, and they would degrade ECM components to facilitate RPs to breach the renal epithelium.

In cardiovascular diseases, increased MMP1 expression in the heart led to a disruption of structural collagen and cardiac dysfunction (30). Activation of MMP3 promoted ECM damage and increased the risk for developing abdominal aortic aneurysms (31). MMP9 and MMP12 promoted vascular smooth muscle cell proliferation *via* beta-catenin pathway and contributed to atherosclerosis (32). MMP10 played a pivotal role in aortic valve calcification by inducing cell mineralization (33). Studies also found the levels of MMP1, MMP3, and MMP12 were positive associated with the degree of atherosclerosis and plaque stability, acting as valuable biomarkers for clinical diagnosis and prognosis of atherosclerosis (34). In gut inflammatory diseases, TNFα induced the expression of MMP1, which remodeled ECM to cause colon damage. MMP1 could also increase the expression of TNFα in a positive feedback mechanism to result in excessive mucosal damage (35). D’Haens et al. have developed 13 blood biomarkers to improve diagnosis of patients with Crohn’s disease, which included MMP1, MMP3, MMP9 (36). In joint inflammatory disease, synoviocytes expressed MMP1, MMP3, and MMP10 to increase their invasiveness to destruct the cartilage in rheumatoid arthritis (37). MMPs were involved in the proteolytic cleavage of important tissue components surrounding the joint and collagen fragments degradation, exposing various immunodominant epitopes for immune response (38). Besides, MMPs participate in cancer progression, metastasis and angiogenesis (9).

There is an increasing focus on the role of MMPs in renal diseases. Serum MMP1 was found to be significantly elevated in polycystic kidney disease (39). MMP3 could mediate shedding of kidney injury molecule-1 in renal tubular epithelial cells during acute kidney injury (40). The upregulation of MMP9 induced endothelial mesenchymal transition of tubular cells to promote kidney fibrosis (41). The increased expression of MMP10 was found specifically in the podocytes of injured glomeruli, further causing proteinuria and glomerulosclerosis (42). However, the relationship between MMPs and urolithiasis has been little investigated. Wu et al. found a high calcium concentration activated the ROS/NF-κB/MMP9 axis in tubular cells to facilitate osteoblastic transformation and crystals deposition (43). Also, MMP9 gene polymorphisms was also found to be related with urolithiasis (44).

We divided 29 RPs samples into two clusters based on the expression of hub DEMMPs. Cluster A exhibited high expression of hub DEMMPs while cluster B exhibited low expression. GO analysis showed DEGs were enriched in leukocyte mediated immunity, leukocyte chemotaxis, myeloid leukocyte migration, suggesting MMPs might participate in RPs progression through immunity and inflammation. KEGG analysis showed DEGs were enriched in inflammatory diseases, such as rheumatoid arthritis, inflammatory bowel disease, and these diseases were confirmed to be related with MMPs. The results of GSEA were also consistent with the above findings, as cluster A was enriched with activation of immune response, acute inflammatory response and adaptive immune response. Thus, we further conducted immune infiltration analysis and found that M1 macrophages were more abundant in cluster A. Markers of M1 macrophages, including NOS2, TLR2, and TLR4, were also higher in cluster A. All hub DEMMPs showed positive correlation tendency with M1 macrophages, although MMP9 and MMP10 did not reach statistical significance. Studies found that mice with a deficiency of MMP3 or MMP9 showed reduced macrophage infiltration in atherosclerosis in atherosclerotic plaques (45, 46). Similarly, our results indicated that increased expression of MMPs was accompanied by more M1 macrophages infiltration.

Macrophage function in CaOx stone formation has been a hot research topic in recent years (13). M0 macrophages can be polarized into two major phenotypes, pro-inflammatory M1 macrophages and anti-inflammatory M2 macrophages based on different conditions. The renal papillary tissues of stone formers exhibited higher expression of M1-related genes and lower expression of M2-related genes (47). Studies have confirmed that M1 macrophages could release pro-inflammatory molecules and induce renal damage and inflammation to promote crystals deposition and stone formation (47–49). Conversely, the M2 macrophages exert protective effects against stone formation owing to their potent capacity to phagocytose crystals (47, 50, 51). Hence, regulation of macrophage phenotypic transformation from M1 to M2 might be therapeutic targets.

Various studies have been conducted to elucidate the relationship between MMPs and macrophages. On the one hand, macrophages are an important source of MMPs, such as MMP9, MMP10, and MMP12. MMP9 from macrophages could promoted elastin degradation and accelerated disruption of atherosclerotic plaques (52). M1 macrophages could release MMP10 to induce pulmonary artery smooth muscle cells proliferation and migration, further leading to vascular remodeling and pulmonary arterial hypertension (53). MMP12, also called macrophage elastase, could mediate a variety of pathological processes. On the other hand, MMPs secreted by other cells can induce macrophage infiltration. For example, MMP9 induced macrophages recruitment through cleavage of SPP1 (54). Several MMPs, including MMP1, MMP3, MMP9, and MMP12, can process proTNF on macrophages to its active form, which might evoke the constitutive release of TNF from macrophages to induce tissue damage (55). We assumed that cluster A might recruit more M1 macrophages around RPs to exacerbate inflammatory responses and promote RPs progression and stone formation.

This study has several limitations. First, our results are mostly based on bioinformatics analysis and the underlying mechanisms behind the role of MMPs in urolithiasis remains unclear, which need to be further verified and clarified by *in vitro* and *in vivo* experiments. second, the sample size of the public dataset was still relatively small, which require further large samples validation.

## Conclusion

In summary, we identified five hub DEMMPs implicated in urolithiasis *via* WGCNA and machine learning algorithms. What’s more, RPs samples were classified into two clusters based on their expression profile. Cluster A which exhibited higher expression of hub DEMMPs is enriched with immune response and inflammation through functional analysis. Besides, cluster A showed higher immune infiltration levels, especially increased infiltration of M1 macrophages and enhanced levels of inflammation compared to cluster B. Our findings offer a novel perspective on the involvement of MMPs in the development of RPs and stone formation for the first time, and provided potential targets for treatment and prevention.

## Data availability statement

The original contributions presented in the study are included in the article/supplementary material. Further inquiries can be directed to the corresponding authors.

## Author contributions

S-YH, S-GW and B-LQ designed the study. S-YH and H-CJ performed the analysis. S-YH, H-CJ and W-CX wrote the manuscript. W-CX and H-SZ contributed to preparing the figures and tables. H-SZ and S-GW revised the manuscript. B-LQ provided the funding and supervised the study. All authors contributed to the article and approved the submitted version.
